# Proanthocyanidins: structure, biosynthesis, regulation, and structure–activity relationships

**DOI:** 10.1016/j.abiote.2026.100047

**Published:** 2026-03-31

**Authors:** Yi Qiao, Pan Zhen, Qian Gao, Feifei Yu, Qi Xie, Jiayang Shi

**Affiliations:** a State Key Laboratory of North China Crop Improvement and Regulation, College of Life Sciences, Hebei Agricultural University, Baoding, 071000, China; b Institute of Genetics and Developmental Biology, The Innovative Academy of Seed Design, Chinese Academy of Sciences, Beijing, 100101, China; c College of Grassland Science and Technology, China Agricultural University, Beijing, 100193, China; d National Center of Technology Innovation for Maize, State Key Laboratory of Crop Germplasm Innovation and Molecular Breeding, Syngenta Group China, Beijing, 102206, China; e Technology Center, Shanxi Xinghuacun Fenjiu Distillery Co., Ltd, Fenyang, 032205, China

**Keywords:** Proanthocyanidins, Condensed tannins, MBW complex, (−)-epicatechin, (+)-catechin

## Abstract

Proanthocyanidins (PAs) are polyphenolic compounds widely distributed throughout the plant kingdom, playing critical biological and ecological roles, including in seed dormancy and defense. Owing to their powerful antioxidant, protein-binding, and antimicrobial properties, PAs also have broad applications in healthcare, the food industry, and animal nutrition. Here, we examine recent advances in PA research, focusing on their structural diversity, biosynthetic pathways, regulatory networks, and potential applications. All PAs are oligomers or polymers of flavan-3-ol monomers. PA biosynthesis begins with the phenylpropanoid pathway and the production of the starter units (−)-epicatechin and (+)-catechin. Polymerization proceeds by the addition of activated extension units through non-enzymatic mechanisms. PA biosynthesis is facilitated by metabolic compartmentation and precisely regulated by the MYB–bHLH–WD40 core transcriptional complex, which integrates signals from various phytohormones and environmental factors. Despite sharing common building blocks, PAs are structurally extremely complex, due to variations in their constituent monomers, degree of polymerization, linkage patterns, and chemical modifications. These structural features of PAs collectively determine their physicochemical properties and biological activities. Future PA research should focus on key knowledge gaps, including the site(s) of polymerization within the cell, the mechanisms that determine polymer structure, and the precise structure–activity relationships of PAs. Combined with advanced purification technologies and gene editing and synthetic biology strategies, this knowledge will allow the precise biomanufacturing of target PAs, thereby advancing both fundamental research and industrial applications.

## 1 Introduction

### 1.1 Definition, chemical structures, and classification of proanthocyanidins

#### 1.1.1 Basic chemical structure and constituent units

Proanthocyanidins (PAs), also known as condensed tannins, are an important class of polyphenolic compounds widely distributed throughout the plant kingdom. Plant-derived PAs are among the most abundant natural aromatic polymers after lignin, accounting for over 90% of the commercial tannin market and annual production exceeding 200,000 tons, highlighting their ecological and economic importance [1]. Grape (*Vitis vinifera*) seeds, walnuts (*Juglans regia*), peanut (*Arachis hypogaea*) skins, cocoa (*Theobroma cacao*) shells, apple (*Malus domestica*) peels, pine (*Pinus pinaster*) bark, and various leguminous plants are all rich sources of PAs [2].

PAs are assembled from flavan-3-ol monomers. The basic skeleton of each monomer consists of an A-ring, a C-ring, and a B-ring. The A-ring typically exhibits a resorcinol-type structure (with hydroxyl groups at positions 5 and 7), possessing strong nucleophilicity. The C-ring is an oxygen-containing heterocycle, and the stereochemistry at the C2 and C3 positions determines whether the monomer is (−)-epicatechin (*cis*) or (+)-catechin (*trans*). The C-ring is saturated, distinguishing it from flavonols. The B-ring has either a catechol-type or pyrogallol-type structure and is the primary contributor to PA antioxidant activity. It can directly donate hydrogen atoms to quench free radicals and stabilizing effects of adjacent hydroxyl groups to form stable semiquinone radicals, thereby terminating oxidative chain reactions. The same B-ring structure may also chelate transition metal ions such as iron and copper, blocking their generation of free radicals and thereby inhibiting oxidation at its source [2].

**Fig. 1:**
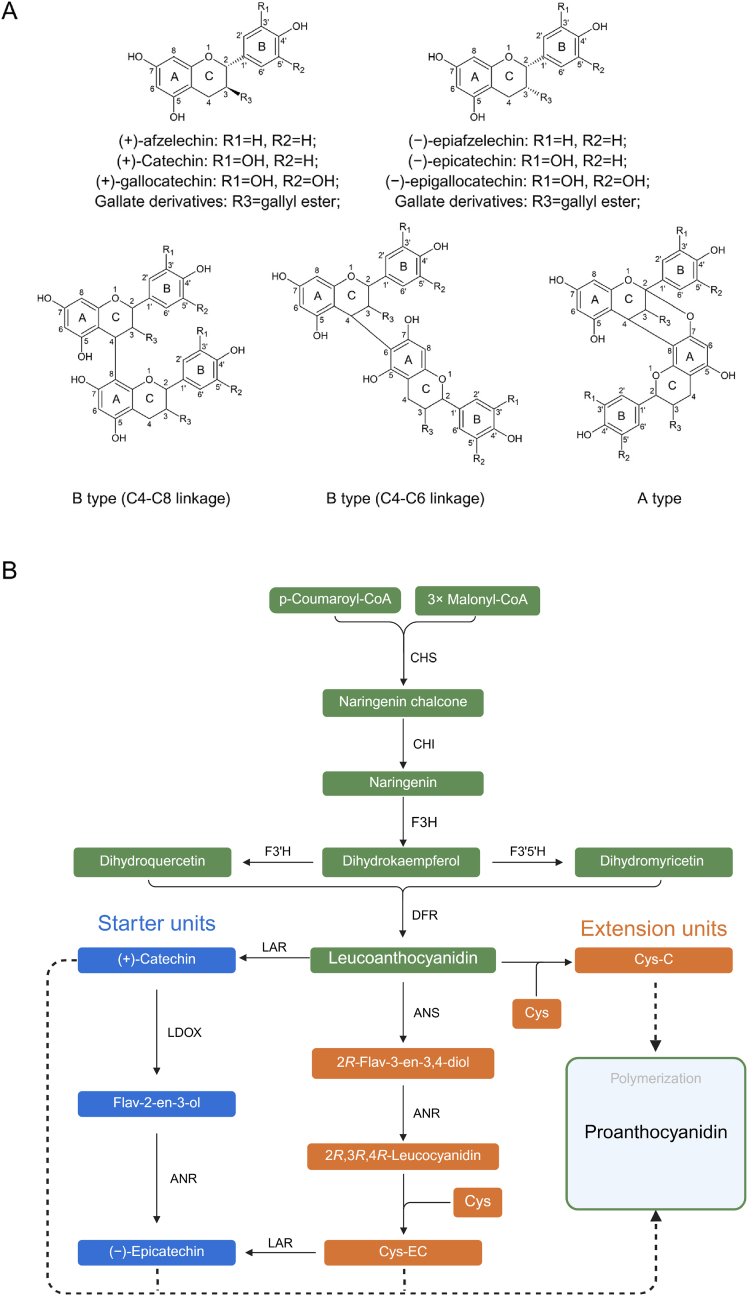
Structures of flavan-3-ols and diagram of the proanthocyanidin biosynthetic pathway. **A** Chemical structures of major flavan-3-ol monomers, including (+)-afzelechin, (+)-catechin, (+)-gallocatechin, and their 2,3-cis or galloylated derivatives. The fundamental interflavan linkage patterns distinguish B-type structures, characterized by single C4 to C8 or C4 to C6 bonds, from A-type structures that feature an additional C2 to O to C7 ether bond. **B** Diagram of the core PA biosynthetic pathway originating from the phenylpropanoid and flavonoid routes. The blue pathway highlights the production of starter units mediated by leucoanthocyanidin reductase (LAR) and anthocyanidin reductase (ANR), while the orange pathway illustrates the formation of activated extension units, such as Cys-C and Cys-EC, which serve as essential precursors for subsequent non-enzymatic polymerization into PAs. C, (+)-catechin; EC, (−)-epicatechin; Cys-C, 4β-(S-cysteinyl)-catechin; Cys-EC, 4β-(S-cysteinyl)-epicatechin; CHS, chalcone synthase; CHI, chalcone isomerase; F3H, flavanone-3-hydroxylase; F3′H, flavonoid 3′-hydroxylase; F3′5′H, flavonoid 3′,5′-hydroxylase; DFR, dihydroflavonol-4-reductase; LAR, leucoanthocyanidin reductase; LDOX, leucoanthocyanidin dioxygenase; ANS, anthocyanidin synthase; ANR, anthocyanidin reductase.

The most common constituent monomers of PAs are (−)-epicatechin and (+)-catechin. Differences in the B-ring hydroxylation pattern give rise to different types of flavan-3-ol monomers. For example, those with hydroxyl groups only at the 4′ position of the B-ring belong to the afzelechin series [(+)-afzelechin or (−)-epiafzelechin]; those with hydroxyl groups at the 3′ and 4′ positions belong to the catechin series [(+)-catechin or (−)-epicatechin]; and those with hydroxyl groups at the 3′, 4′, and 5’ positions belong to the gallocatechin series [(+)-gallocatechin or (−)-epigallocatechin]. Furthermore, the C3 hydroxyl group of flavan-3-ols can undergo galloylation (attachment of a gallic acid moiety), forming derivatives such as epigallocatechin gallate and further increasing PA structural complexity and biological activity (Fig. 1A) [3].

#### 1.1.2 Polymerization patterns and structural diversity

PA diversity arises from monomer heterogeneity and differences in the way the monomers are joined together. PA monomers are primarily connected through C4→C8 or C4→C6 bonds, termed B-type linkages. A-type linkages are formed when two units are simultaneously connected by both C4→C8 (or C4→C6) and C2→O→C7 (or C2→O→C5) bonds (Fig. 1A) [4,5]. The proportion of A-type bonds varies greatly among PAs from different plant sources; for instance, only about 5% of apple PAs contain A-type bonds, while this proportion can reach 96% in cranberry (*Vaccinium macrocarpon*) [6]. The mean degree of polymerization (mDP) of PAs is a key parameter determining their physicochemical properties such as astringency and protein-binding capacity [7,8]. The mDP varies extensively across different plant tissues at different developmental stages [8].

#### 1.1.3 Differences in structure and distribution between PAs and other tannins

Although they are grouped with “tannins”, PAs differ fundamentally in their chemical structure from hydrolyzable tannins and phlorotannins; they possess distinct physicochemical properties, originate from different biosynthetic pathways, and have different species distributions.

Hydrolyzable tannins (molecular weight 500–3000 Da) are oligomers formed through esterification of a carbohydrate core (usually d-glucose or similar polyols) with phenolic acids. Hydrolyzable tannins can be further subdivided into gallotannins and ellagitannins. Gallotannins possess a central sugar unit that is partially or fully esterified with gallic acid. Ellagitannins consist of gallic acid and at least one hexahydroxydiphenoyl unit linked to a monosaccharide core, primarily glucose [1,9]. Plant families rich in ellagitannins (with crude extract content >90 mg/g) include *Onagraceae*, *Gentianaceae*, *Rosaceae*, *Elaeagnaceae*, and *Fagaceae* [10].

Phlorotannins are polyphenolic oligomeric and polymeric structures formed from phloroglucinol monomers. These compounds are primarily biosynthesized in the Golgi apparatus of brown algae. Phlorotannins are highly water-soluble, with molecular weights ranging from 126 to 650 kDa. To date, approximately 150 phlorotannins have been identified [11].

### 1.2 Biological functions and ecological significance of PAs

The biological and ecological functions of PAs are a consequence of their accumulation in specific tissues and developmental stages. These functions primarily stem from their chemical properties, particularly their ability to interact with proteins, metal ions, and other molecules [1,12].

PAs play multiple roles in seed physiology, including in seed longevity and the timing of germination. In Arabidopsis (*Arabidopsis thaliana*), *transparent testa* (*tt*) mutants defective in PA biosynthesis and regulation exhibit weaker seed dormancy than their corresponding wild types. Indeed, nine days after seed harvest, the germination rate of wild-type seeds is essentially zero, while that of *tt11* and *transparent testa glabra 1* (*ttg1*) mutant seeds reaches 100%. Simultaneously, seed longevity is significantly lower in these mutants, which have higher seed coat permeability, indicating PA involvement in maintaining seed dormancy [13]. Additionally, PA biosynthesis is closely associated with normal development of the seed coat cuticle. In the ii1 cell layer of Arabidopsis seed coat, PA biosynthesis is essential for deposition of cuticle-related substances. In *tt* mutants, abnormal cuticle development and higher permeability indicate that PA accumulation is necessary for forming a complete seed coat and thus a physicochemical barrier surrounding the seed [14].

PAs also confer resistance biotic stress. Sorghum (*Sorghum bicolor*) varieties that accumulate PAs exhibit significant resistance to birds feeding, likely due to the astringent taste conferred by PAs [15]. When black poplar (*Populus nigra*) trees are infected with the fungus *Melamspora larici-populina*, (+)-catechin accumulation in leaves increases significantly; silencing the PA pathway transcription factor gene *MYB134* diminishes PA concentration by 50% and raises infection severity by 50% [16]. Furthermore, PA oligomers extracted from pine bark exhibit broad-spectrum antifungal activity against various yeasts (such as *Candida*, *Cryptococcus*) [17].

In addition to their roles in plant physiology, PAs have a significant impact on forage quality. The protein-rich contents of important forage legumes such as alfalfa (*Medicago sativa*) and white clover (*Trifolium repens*) rapidly ferment in the rumen of ruminant animals. This fermentation produces large amounts of gas and foam, leading to potentially fatal bloat. PAs can complex with the forage proteins and the bacterial enzymes that ferment them, thereby significantly lowering the rate of protein fermentation in the rumen. In this way, PA accumulation in protein-rich forage crops prevents bloat and allows more of the protein to be absorbed in the intestine, thereby improving milk and meat production [[18], [19], [20]].

### 1.3 Research background and review objectives

PA research has progressed from early genetic screens and individual gene identification to gradually establishing the core framework of PA biosynthesis, regulation, and transport. Studies of Arabidopsis *tt* mutants have identified key biosynthetic enzymes such as anthocyanidin reductase (ANR) and leucoanthocyanidin reductase (LAR), along with regulatory factors including TT2, TT8, and TTG1, establishing a molecular foundation for this field. At the same time, the inherent complexity of this framework has gradually emerged, with many core mechanistic questions remaining open.

Although the biosynthetic pathways of PA constituent monomers are relatively well understood, several critical gaps remain. These include the specific polymerization steps and transport mechanisms, the integration of existing models of PA compartmentalization and vacuolar storage, the mechanisms by which the conserved MBW regulatory network achieves precise control of PA content, composition, and degree of polymerization across different species, tissues, and developmental stages, and the relationships between PA structural diversity and specific biological activities. Addressing these gaps is essential for advancing the transition from crude plant extracts to high-value products with defined PA structures in medicine, food, feed, and related fields. This review systematically examines recent advances in PA research, focusing on three major topics: 1) the conserved and divergent features of PA biosynthetic pathways across species; 2) the intricate regulatory networks that control PA accumulation; and 3) the prospects for diversified applications based on the structure–activity relationships of PAs. The goal is to clarify the key remaining scientific questions concerning PAs and to advance both fundamental research and industrial applications of PAs to new levels.

## 2 PA biosynthetic pathways

### 2.1 Core biosynthetic pathways

#### 2.1.1 Upstream pathway: from phenylpropanoids to anthocyanidins

PA biosynthesis begins with the phenylpropanoid pathway. In this pathway, phenylalanine undergoes deamination by l-phenylalanine ammonia-lyase (PAL) to yield *trans*-cinnamic acid which is then hydroxylated into *p*-coumaric acid by cinnamate-4-hydroxylase (C4H), after which *p*-coumaric acid is conjugated with coenzyme A via catalysis by 4CL to form *p*-coumaroyl-CoA. P-coumaroyl-CoA, serving as a starter unit, condenses with three molecules of malonyl-CoA under catalysis by chalcone synthase (CHS) to form naringenin chalcone. Naringenin chalcone is isomerized into naringenin by chalcone isomerase (CHI).

Naringenin enters the core flavonoid metabolic pathway and is converted to dihydrokaempferol (DHK) by flavanone-3-hydroxylase (F3H). DHK can be further converted to dihydroquercetin (DHQ) or dihydromyricetin (DHM) by flavonoid 3′-hydroxylase (F3′H) or flavonoid 3′,5′-hydroxylase (F3′5′H), respectively; DHK, DHQ, and DHM are collectively termed dihydroflavonols.

Dihydroflavonols represent a key branch point in the flavonoid pathway, with their metabolic fate determining the production of either anthocyanins and PAs or flavonols. Specifically, the dihydroflavonol-4-reductase (DFR) pathway leads to leucoanthocyanidins, which are oxidized by anthocyanidin synthase (ANS) to form 2*R*-flav-3-en-3,4-diol. This key intermediate is the direct precursor for PA biosynthesis and can also be glycosylated to form stable pigments like cyanidin-3-*O*-glucoside [21]. By contrast, the flavonol synthase (FLS) pathway converts dihydroflavonols into various flavonols, such as kaempferol and quercetin (Fig. 1B).

#### 2.1.2 Production of starter units by parallel pathways mediated by LAR and ANR

The starter units for PA polymerization are primarily (+)-catechin and (−)-epicatechin and are produced through two key enzymatic pathways. These two pathways share upstream precursors but exhibit significant differences in their enzymatic mechanisms and species distribution. The relative activities of these pathways jointly fine-tune PA composition.

One core pathway begins with LAR, which reduces leucoanthocyanidins produced by DFR to generate (+)-catechin [22]. This pathway is relatively direct, providing in one step 2,3-*trans* configuration starter units for PA polymerization. However, *LAR* genes are not universally present across the plant kingdom. For example, functional LAR homologs are absent in *Arabidopsis* [21], and PAs in the seed coat of this plant are composed of (−)-epicatechin.

The other core pathway involves ANR and is more complex. Early studies suggested that ANR converts cyanidin to catechin and epicatechin [23,24]. However, subsequent research revealed that the main substrate for ANR is flav-2-en-3-ol, which, at pH 7.0, is produced from (+)-catechin by leucoanthocyanidin dioxygenase (LDOX). ANR can also act on cyanidin, an oxidation product of flav-2-en-3-ol, but less efficiently. In both cases, the product is (−)-epicatechin [21,22]. Under acidic conditions (pH 5.0), incubation of cyanidin with AtANR results in the partial reduction of cyanidin to flaven-2-en-3-ol, which may explain how plants lacking the LAR/LDOX pathway, such as Arabidopsis, can produce PA starter units (Fig. 1B) [21].

#### 2.1.3 Production of extension units by non-enzymatic polymerization mechanisms

Besides starter units, PA oligomerization also requires activated extension units. The extension of PA chains is primarily completed through non-enzymatic mechanisms, largely based on two key types of thioether adducts: 4β-(S-cysteinyl)-epicatechin (Cys-EC) and 4β-(S-cysteinyl)-catechin (Cys-C).

The supply of extension units for PA polymerization is subject to precise synergistic regulation by ANR and LAR. Specifically, ANR catalyzes the production of (−)-epicatechin starter units and also converts the ANS product 2*R*-flav-3-en-3,4-diol to 2*R*,3*R*,4*R*-leucocyanidin, which forms Cys-EC through a non-enzymatic conjugation reaction with cysteine, proceeding via a (−)-epicatechin carbocation intermediate [21,22]. Therefore, ANR simultaneously regulates the supply of both (−)-epicatechin starter units and Cys-EC extension units. The generation of the Cys-C extension unit is more direct; *in vitro* studies showed that Cys-C can be produced non-enzymatically when leucoanthocyanidin is co-incubated with cysteine (Fig. 1B) [21].

Recent research has uncovered another potential pathway, with the finding that a drop in ascorbic acid (AsA) biosynthesis leads to lower leucoanthocyanidin content. AsA can form AsA-[C]_n_ adducts with leucoanthocyanidins non-enzymatically under *in vitro* conditions. *In vivo*, AsA-[C]_n_ adducts are thought to serve as a more important pool of extension units than Cys-C, participating in non-enzymatic PA polymerization. These findings provide a new perspective on the possible sources of extension units [25].

ANR and LAR indirectly yet effectively control the mDP of PAs through bidirectional regulation of the starter unit/extension unit ratio. ANR promotes polymerization by generating Cys-EC extension unit precursors, providing the “raw materials” for polymerization reactions. LAR, besides catalyzing the production of (+)-catechin starter units, can also convert Cys-EC extension units to (−)-epicatechin starter units in the reciprocal reaction, which diminishes the extension unit/starter unit ratio, thereby favoring chain termination and promoting oligomer formation. The *lar* mutant of barrel clover (*Medicago truncatula*) accumulates lower levels of starter units, leading to the production of more insoluble high-molecular-weight PAs [22,26].

*In vitro* experiments confirm that PA polymerization is a non-enzymatic reaction [22]. At pH > 6.5, the C–S bond at the 4β position in the Cys-EC molecule undergoes heterolysis, generating an active epicatechin carbocation. This carbocation is a powerful electrophile that can attack electron-rich nucleophilic sites (such as the C8 or C6 position) on the aromatic ring of another PA molecule (starter unit or extending chain terminus), forming new C–C bonds (B-type linkages) and extending the chain. This mechanism is supported experimentally: co-incubating epicatechin with Cys-EC *in vitro* at an appropriate pH produces detectable levels of PA trimers and tetramers [22]. Flavan-3-ol carbocations have been directly detected in plant extracts, supporting the existence of this reaction *in planta* [27].

#### 2.1.4 Multi-enzyme complexes and subcellular localization of biosynthetic enzymes

Efficient PA biosynthesis depends on sequential enzymatic reactions and benefits from the precise spatial organization of the corresponding enzymes within cells. Enzymes in the PA biosynthetic pathway may work synergistically by forming “metabolic compartments” or “multi-enzyme complexes,” an organizational strategy that helps improve substrate channeling efficiency and prevent exposure of cells to potentially toxic intermediates.

In grape, ANS and ANR are primarily located in the cytoplasm [28,29]. This overlapping localization may facilitate the direct utilization of ANS products as substrates by ANR, increasing the efficiency of converting anthocyanidins into PA starter units.

More compelling results comes from studies of enzymes further upstream in the flavonoid pathway. In Arabidopsis root cells, CHS and CHI co-localize in electron-dense particles. Notably, this co-localization disappears in mutants lacking F3′H, which is typically anchored in the endoplasmic reticulum (ER) membrane [30,31]. This finding strongly suggests the existence of a multi-enzyme complex at the ER membrane. This hypothesis is supported by data in soybean (*Glycine max*): ER membrane-localized isoflavone synthase (IFS) and C4H can recruit the downstream soluble enzymes CHS, chalcone reductase (CHR), and CHI to the ER surface, forming an efficient “metabolic compartment” [32,33].

In *Arabidopsis*, key branch point enzymes of the flavonoid pathway, such as FLS1 and DFR, interact with CHS in a competitive manner [34]. This interaction may be an important regulatory mechanism, controlling substrate flow to different branches to finely tune end-product ratios.

In summary, PA biosynthesis is not completed by free enzymes dispersed randomly in the cytoplasm but proceeds efficiently through highly ordered enzyme complexes in specific subcellular regions (such as cytoplasmic regions near the ER). This spatial organization establishes the foundation for rapid conversion of precursors and regulation of metabolic flux.

#### 2.1.5 Transport and compartmentalization: from biosynthesis sites to vacuolar storage

PAs in plant cells accumulate in specific compartments; however, the mechanisms governing their transport and the site and mechanism of their final polymerization are still debated. Distinct pathways are proposed by the two primary models: the tannosome-shuttle model and the membrane transporter model.

Tannosome-Shuttle Model. In this model, PA biosynthesis begins inside chloroplasts, with PA polymerization taking place in organelles called tannosomes. Tannosomes originate from the chloroplast thylakoid membrane, which fragments to form spherical bodies approximately 30 nm in diameter. PAs accumulate inside the tannosomes and when they reach a given threshold, the tannosomes become enveloped by a membranous vesicle formed from the fusion of the inner and outer chloroplast membranes; this vesicle is called a shuttle. Shuttles then bud off from the chloroplast outer envelope and enter the vacuole, where they do not immediately dissolve but rather aggregate, forming large tannin deposits visible under light microscopy. Therefore, the long-observed tannin deposits within vacuoles are composites formed by countless shuttles filled with PAs and originating from chloroplasts [35,36]. This mechanism is conserved across various vascular plant species (such as grape, spruce [*Picea asperata* Mast.], and persimmon [*Diospyros kaki*]), explaining the deposition of PAs within their vacuoles [36]. Ripe pear fruits (*Pyrus communis*) accumulate PAs inside the vacuoles of large parenchyma cells, where they appear as extremely dense, uniformly stained spheres (0.5–1 μm in diameter); these particles may be shuttles [37]. However, research on the tannosome-shuttle model remains limited. Furthermore, how this model integrates with the membrane transporter–based model described below has yet to be resolved.

Membrane transporter–based model. In this model, PA precursors enter the vacuole via transporters in the tonoplast membrane and polymerization occurs inside the vacuole. Before transport, unstable PA precursors typically require glycosylation to improve stability. For example, in *M. truncatula*, the UDP-glucosyltransferase UGT72L1 can catalyze the production of epicatechin-3′-*O*-glucoside (E3′G) from epicatechin [38]. PA transport exhibits significant specificity. Multidrug and Toxic Compound Extrusion (MATE)-type family transporters such as AtTT12 in *Arabidopsis* and MtMATE1 in *M. truncatula* recognize and transport specific glycosylated substrates from the cytoplasm across the vacuolar membrane; they transport E3′G with a higher affinity and rate than anthocyanin-3-*O*-glucoside (Cy3G) but cannot effectively transport catechin-3-*O*-glucoside (C3G) or flavonol glucosides into the vacuolar lumen [39].

AtTT13, also reported as the proton ATPase AHA10, uses the energy derived from ATP hydrolysis to pump protons (H^+^) into the vacuole. This activity maintains the acidic environment of the vacuolar lumen and provides the proton motive force needed for secondary active transport mediated by MATE transporters. The specific expression of *TT13* in the seed coat endothelium of *Arabidopsis*, coinciding with PA accumulation sites, is consistent with its dedicated role in PA transport [40].

The correct localization of transporters to the vacuolar membrane is a prerequisite for their function. In plants ranging from *Arabidopsis* and *M. truncatula* to tea plants (*Camellia sinensis*) and cotton (*Gossypium hirsutum*), proteins homologous to TT12 all localize to the vacuolar membrane [38,39,41,42]. In tea plants, phosphorylation of the Ser-17 residue of CsTT12 is crucial for its stable localization to the vacuolar membrane. When this site is mutated to a nonphosphorylatable amino acid, CsTT12 is mis-localized and primarily accumulates in the cytoplasm, leading to functional impairment [42]. Thus, transporter activity may be regulated by post-translational modifications such as phosphorylation.

Mutation of *UGT72L1* in *M. truncatula* resulted in 50% lower content of soluble PAs (i.e., the fraction extractable by organic solvents and amenable to standard analysis methods) than in the wild type, while the levels of insoluble PAs remained unchanged [43]; in an *Arabidopsis* *tt12* mutant, soluble PAs were almost undetectable, while the content of insoluble PAs dropped dramatically to 15% of that of the wild type [39]. In *Arabidopsis*, the level of insoluble PAs far exceeds that of soluble PAs, suggesting that highly polymerized PA formation may have a separate, E3′G-independent starter or extension mechanism. TT12 may also transport other substrates critical for PA formation that have yet to be identified. The membrane transporter–based model has yet to be integrated with the Cys-EC biochemical model, as it does not account for the transport of the Cys-EC extension units into the vacuole.

### 2.2 Variation in PA biosynthetic pathways across species

A comparative analysis of PA structures across multiple plant species reveals that their biosynthetic pathways, while built on a conserved framework, exhibit significant species variation (Table S1). This variation is primarily manifested at three levels—constituent units, polymerization architecture, and chemical modifications—which collectively determine PA physicochemical properties and functional specificity.

Although (−)-epicatechin and (+)-catechin are the most prevalent building blocks for PA biosynthesis, there are differences in B-ring hydroxylation patterns (such as whether gallocatechin units are present) and monomer configuration (*cis* or *trans*). For example, PAs in species like yellow-puff (*Neptunia lutea*) contain abundant (+)-gallocatechin units, while PAs in *M. truncatula* consist almost entirely of (−)-epicatechin. Such differences in monomer composition directly arise from variation in *F3′H*, *F3′5′H*, *LAR*, and *ANR* expression levels and enzyme activities [44,45]. Diversity in polymerization architecture is even greater: mDP varies dramatically among species and even between different tissues of the same species, such as berry skin and seeds in grape, or leaves and branches in blueberries (*Vaccinium corymbosum*). PAs can accumulate as oligomers (such as in plum, with mDP ∼5) or highly polymerized forms (such as in black chokeberry [*Aronia melanocarpa*], with mDP ∼49) [46,47]. Inter-monomer linkage patterns (A-type or B-type) also show strong species preferences; for instance, A-type linkages dominate (>96%) in cranberry and peanuts, while B-type linkages are more prominent in apple and cocoa [6,48]. These differences in linkage patterns are a key factor driving the molecular conformations and biological activities of distinct PAs. Finally, whether and to what extent the chemical modification of galloylation at the 3-*O*-position occurs is another important source of variation. For example, over 70% of all PAs in persimmon are galloylated, while many other species show very low levels or no detectable galloylation [49]. In summary, PA biosynthetic pathways show extensive species-specific variation in structure. Although this diversity suggests evolutionary adaptation to different ecological pressures, direct evidence linking specific PA structures to adaptive advantages in most species requires further investigation. Understanding the patterns of PA structural variation and how they arise will form the foundation for future targeted improvement of crop quality or screening for PAs with specific functions.

### 2.3 Evolution and functional divergence of key biosynthetic genes

PA biosynthesis is built upon flavonoid metabolism. The *CHS* family, encoding the enzymes that catalyze the first committed step of the flavonoid pathway, dates back to the common algal ancestor of the green plant lineage and may have played crucial roles in early adaptation during the transition from aquatic to terrestrial environments [50]. There are four types of CHI enzymes, of which types I and II are mainly present in vascular plants, type III is found in both land plants and green algae, and type IV is currently believed to exist only in land plants, indicating complex functional diversification of the *CHI* family before and after plant terrestrialization [51].

DFR is a core branch point in the flavonoid pathway, catalyzing the conversion of the dihydroflavonols DHK, DHQ, and DHM to their corresponding leucoanthocyanidins. DFR substrate specificity is determined by a key domain of 26 amino acids, based on which this enzyme can be classified into several types: Asn-type DFRs possess a broad substrate specificity, able to use DHK, DHQ, and DHM as substrates; Asp-type DFRs preferentially use DHQ and DHM as substrates; and additional types such as Arg-type DFRs also exist, which preferentially utilize DHK and DHQ as substrates. Importantly, phylogenetic analysis revealed that Asn-type and Asp-type DFRs coexist in gymnosperms such as ginkgo (*Ginkgo biloba*) and dawn redwood (*Metasequoia glyptostroboides*). This finding indicates that the evolutionary diversification of DFR substrate selectivity predated gymnosperm radiation [52].

ANR exists in both gymnosperms and angiosperms, among which its catalytic core, consisting of an NADPH-binding domain and a substrate-binding pocket, is highly conserved. ANR activity has also been detected in ferns, indicative of the existence of the ANR pathway in seedless vascular plants [53].

Phylogenetic analysis indicates that plant LARs can be divided into two major clades: one clade contains all the LARs of dicotyledonous species, while the other clade contains those of gymnosperms and monocotyledonous species. Thus, monocot LARs are evolutionarily closer to those of gymnosperms than to those of dicots. Within dicots, legume LARs form an independent monophyletic group [54].

The distribution and phylogeny of the genes encoding key enzymes of the PA biosynthetic pathway follow clear evolutionary patterns. The pathway-initiating enzyme CHS has the earliest origin, traceable to algae; the key enzymes for PA biosynthesis, ANR and LAR, are present in both gymnosperms and angiosperms, indicating that the complete PA biosynthetic pathway was already established in their common ancestor.

## 3 Regulatory networks for PA accumulation

### 3.1 Universal regulation by core MBW transcriptional regulatory complexes

In *Arabidopsis*, PAs accumulate in specific cell layers of the seed coat, and their biosynthesis is primarily controlled by a ternary transcriptional activation complex comprising R2R3-MYB and bHLH transcription factors together with a WD40 repeat–containing protein (WD40), forming an MBW complex [[55], [56], [57]].

Several core components of the MBW complex were identified via *tt* mutants. For example, AtMYB123, also named TT2, is a typical R2R3-MYB transcription factor belonging to the subgroup 5 MYB clade and is the primary regulator of PA biosynthesis in *Arabidopsis* [58,59]. Loss of TT2 function leads to much lower expression levels for PA biosynthetic genes, including *DFR*, *ANS*, *ANR*, *TT12*, *TT19*, and *AHA10*, thereby preventing PA accumulation [57].

The bHLH cofactors TT8 and ENHANCER OF GLABRA 3 (EGL3) interact with TT2 to co-activate expression of their downstream target genes. Genetic analysis indicates TT8 and EGL3 are functionally redundant in PA biosynthesis, as both the TT2–TT8–TTG1 and TT2–EGL3–TTG1 complexes can activate transcription from downstream genes. However, the other bHLH member, GL3, cannot substitute for TT8 function [55,57,60].

The WD40 protein TTG1 serves as a stabilizing scaffold for the MBW complex. Experiments in protoplasts isolated from the moss *Physcomitrium patens* demonstrated that while co-transfection of *TT2* and *TT8* only weakly activates the promoter of *BANYULS* (*BAN*, an *ANR*), strong activation occurs when TT2, TT8, and TTG1 are present, underscoring the critical role of TTG1 in stabilizing the MBW complex [61]. This complex directly regulates genes encoding the downstream enzymes in the PA pathway, such as *DFR*, *ANS*, *ANR*, *TT19*, *TT12*, and *AHA10*, while regulation of the upstream biosynthetic genes *CHS*, *CHI*, *F3H* is independent of the MBW complex [57].

The MBW complex binds to specific *cis*-acting elements in the promoter regions of target genes. In the promoter of *ANR* in *Arabidopsis*, the MYB core binding site (CTGTTG) and the bHLH binding site (the G-box CACGTG), are both essential, as mutation of either motif completely abolishes transcriptional activation by the MBW complex [57,61].

The regulation of PA biosynthesis by MBW complexes is remarkably conserved throughout the plant kingdom. Homologous MBW complexes have been confirmed as core regulators of PA biosynthesis in strawberry (*Fragaria* × *ananassa*; FaMYB9/11–FabHLH3–FaTTG1) [62], apple (MdMYB9/11–MdbHLH3–MdTTG1) [63] tea plant (CsMYB1–CsGL3–CsWD40) [64], and banana (*Musa acuminata*; MaMYBPA1–MaMYC–MaTTG1) [65].

The key determinants of target gene specificity reside in the R2R3 domain of the MYB component. Domain swapping experiments between the anthocyanin regulatory factor MYB114 and the PA regulatory factor TT2 demonstrated that the R2R3 domain dictates promoter targeting specificity in *Arabidopsis*. Amino acid residue 39, a glycine in the R2 repeat, and amino acids 90–93 (DNEI) in the R3 repeat, are critical for determining the specific activation of *ANR* by TT2 [66].

In *Arabidopsis*, functional redundancy ensures regulatory robustness. While TT2 is the primary PA regulator, AtMYB5 serves as a secondary factor; indeed, expression of *DFR* and *ANS* was nearly undetectable in *tt2 myb5* double mutant plants. In the *tt2* single mutant, DFR and ANS expression was only barely detectable in the endothelium, suggesting that TT2 plays the predominant role while MYB5 plays a supporting function (TT2 is expressed in the micropyle, chalaza, and endothelium, while MYB5 is expressed primarily in the endothelium) [57]. Similarly, the bHLH factors TT8 and EGL3 are functionally redundant, although the expression patterns of their encoding genes show some spatial specificity (*TT8* is expressed in the endothelium, *EGL3* in the chalazal region), which may contribute to fine-tuning PA distribution in different parts of the seed [57].

### 3.2 Other regulatory networks

**Fig. 2:**
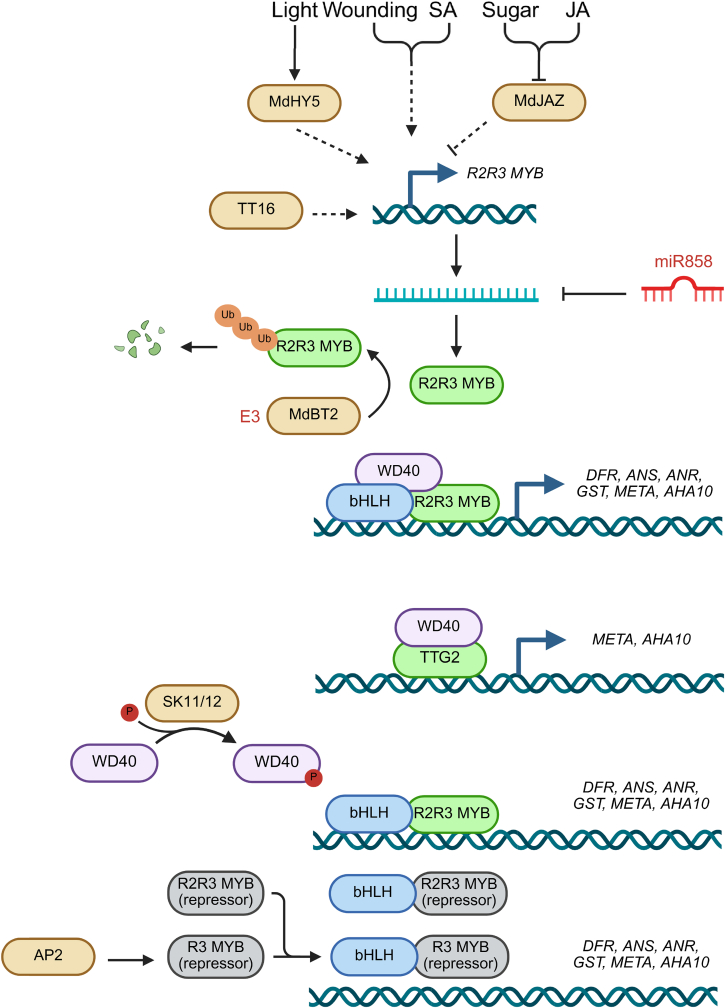
A multilayer regulatory network governing proanthocyanidin biosynthesis across plant species. The accumulation of PAs is orchestrated by a complex regulatory network that integrates environmental stimuli, phytohormonal signals, and transcriptional control. Upstream signals such as light, mechanical wounding, and endogenous factors such as sugar and phytohormones (e.g., jasmonic acid [JA] and salicylic acid [SA]), modulate the expression of R2R3-MYB genes, often involving intermediaries such as HY5 or JAZ repressor proteins. Central to this network is the ternary complex comprising R2R3-MYB, bHLH, and WD40 proteins (MBW), which directly activates the expression of downstream PA biosynthetic genes and transport-related genes such as *TT12* and *AHA10*. This activation is further fine-tuned by multiple inhibitory layers, including post-transcriptional mRNA cleavage by miR858, ubiquitin–proteasome degradation mediated by the E3 ubiquitin ligase BT2, and competitive binding from R3-MYB or R2R3-MYB repressors, while transcription factors TTG2 and TT16 ensure PA biosynthesis is restricted to appropriate tissues and cell types (such as specific seed coat layers) and that cellular conditions are optimal for PA synthesis and vacuolar storage. JA, jasmonic acid; SA, salicylic acid; HY5, LONG HYPOCOTYL 5; JAZ, JASMONATE ZIM-DOMAIN PROTEIN; TT12, TRANSPARENT TESTA 12; AHA10, H^+^-ATPASE 10; BT2, BTB and TAZ domain protein 2; TTG2, TRANSPARENT TESTA GLABRA 2.

The MADS-box transcription factor TT16 does not directly regulate the expression of PA biosynthetic genes but does control the correct spatial expression of *TT2* by determining inner integument cell fate. In *tt16* mutants, the expression of TT2 is restricted to the micropyle region, whereas in wild-type plants TT2 expression extends throughout the entire endothelium, preventing PA accumulation in the endothelium; ectopic expression of TT2 driven by the p70S promoter (p70S::TT2) can restore PA accumulation in *tt16* mutant seeds demonstrating that TT16 acts upstream of *TT2* (Fig. 2) [67,68].

The WRKY-type transcription factor TTG2 directly activates the expression of the transporter genes *TT12* and *TT13* but requires physical interaction with TTG1 to form an active transcriptional complex [69]. In grape, the TTG2 homolog VvWRKY26 simultaneously activates the expression of PA biosynthetic genes (*ANR*, *LAR1*) and that of vacuolar acidification genes encoding V-ATPase subunits (*PH1* and *PH5*), synergistically optimizing PA biosynthesis and storage, respectively [70].

R2R3-MYB transcriptional activators regulating the PA biosynthesis pathway can be divided into three major categories. The first category is TT2-like (*Arabidopsis* TT2 type), represented by *Arabidopsis* TT2 (also named AtMYB123). These MYBs are the primary regulatory factors for PA biosynthesis, especially in seeds. TT2-type homologs are highly conserved and widely distributed across diverse plant species, including legumes, woody plants, and various crops [16,[71], [72], [73], [74], [75], [76], [77], [78], [79], [80], [81]]. The second category is MYB5-type, whose members are conserved across multiple plant species and play diverse roles in regulating PA biosynthesis, including the promotion of *LAR*, *ANR*, and *F3′H* expression in response to developmental and environmental cues. [74,82,83]. MYB5-type members can also regulate other processes besides PA biosynthesis; for instance, MYB6 in Peking poplar (*Populus tomentosa*) promotes PA biosynthesis while inhibiting secondary cell-wall formation through an interaction with KNOX transcription factor (KNAT7) [84]; and FhMYB5 in freesia (*Freesia hybrida*) can activate the expression of upstream and downstream flavonoid biosynthetic genes [85]. The third category of R2R3-MYB transcriptional activators is MYBPA-type, exemplified by VvMYBPA1 in grape [7]. Notably, this family is significantly expanded in dicots with high tannin content but is absent in monocots and basal dicots [82]. This type is also widely distributed, including PpMYBPA1 in peach (*Prunus persica*), DkMyb4 in persimmon, MYBPA1.1 in blueberry, and MYB115 in poplar [73,[86], [87], [88]].

R2R3-MYB transcriptional repressors, such as VvMYBC2-L1 and VvMYBC2-L3 in grape, typically contain C-terminal C1 and C2/EAR repressive motifs [89,90]. They function primarily through two mechanisms: competitive inhibition of the binding of activator-type MYBs to their bHLH cofactors, or active repression by recruiting co-repressors through their EAR motifs. For example, PpMYB18 can compete with PpMYBPA1 for binding to PpbHLH3 and actively repress transcription through its C-terminal motif [88]. In black cottonwood, buckwheat, and various other plant species, transcriptional repressors can repress the expression of late PA biosynthetic genes through their interactions with bHLHs [[91], [92], [93], [94], [95]]. Notably, the expression patterns of these repressor factor genes also exhibit fine spatiotemporal tuning. For example, the expression of *MtMYB2* is repressed in the hilum region of seeds, forming a signal gradient that enables PA biosynthesis to begin first in the hilum, chalaza, and micropyle regions, before gradually expanding throughout the entire seed body [96].

R3-MYB type repressor factors such as AtMYBL2 can inhibit PA accumulation in *Arabidopsis* by directly interacting with the MBW complex [97]. Functionally similar factors, such as MYBR3.1 and MYBR3.2, have been identified in blueberry and other species [73].

The MADS-box factor APETALA 2 (AP2) directly binds to the promoter of the repressor gene *MYBL2* and activates its transcription. Subsequently, AP2 forms non-functional complexes with MYBL2 and TT8, thus disrupting MBW activity and inhibiting *ANR*, *TT12*, *TT19*, and *AHA10* expression and suppressing PA biosynthesis [98].

Stability of the MBW complex is subject to dynamic regulation. For example, TTG1 can be phosphorylated by SHAGGY-like kinases (SK11, SK12), thus preventing interaction of TTG1 with TT2; this blocks PA biosynthesis and promotes fatty-acid accumulation during embryo development [99]. BTB and TAZ domain protein 2 (MdBT2) in apple and FaBT2 in strawberry can degrade their respective activators MdMYB9 and FaMYB5 through the ubiquitin–proteasome pathway, thereby negatively regulating PA biosynthesis [83,100].

PA biosynthesis is also regulated by the microRNA miR858, which is widely distributed among angiosperms and can specifically target and cleave mRNAs encoding R2R3-MYBs. In *Arabidopsis*, miR858 negatively regulates PA accumulation by inhibiting *TT2* expression [101]. In cotton, GhmiR858 negatively regulates PA accumulation in leaves and callus by targeting *GhTT2L* transcripts [102]. In apple, the light-responsive factor B-box 22 (MdBBX22) can induce *mdm-MIR858* expression; the mature miR858, in turn, inhibits expression of *MdMYB9*, *MdMYB11*, and *MdMYB12*, redirecting metabolic flux away from PAs and toward anthocyanins [103]. In persimmon, miR858b abundance increases during late fruit development, inhibiting *DkMYB19* and *DkMYB20* expression, thereby suppressing PA accumulation [104]. Thus, the regulation of PA accumulation by miR858-mediated regulatory modules appears to be conserved in seed plants.

### 3.3 Cross-talk between plant hormone and environmental regulatory networks

PA biosynthesis responds to both internal and external signals, which are integrated via a complex network of regulatory cross-talk. The internal signals consist primarily of the phytohormones jasmonic acid (JA) and salicylic acid (SA). External signals include light, wounding, and (indirectly) sucrose levels (Fig. 2).

JA signaling plays a complex and species-specific role in the regulation of PA biosynthesis, mediated through interaction of JASMONATE ZIM-DOMAIN PROTEIN (JAZ) repressor proteins with MBW complex components [105]. In strawberry fruits, JA signaling promotes anthocyanin biosynthesis while inhibiting PA biosynthesis; suppressing JA signaling relieves inhibition of PA biosynthesis, tilting metabolic flux toward the PA branch and thus promoting PA accumulation [106]. In apple, treatment with methyl jasmonate (MeJA) can significantly promote PA accumulation. Indeed, MeJA can strongly induce *MdMYB9* and *MdMYB11* expression. In the presence of JA, JAZ proteins are recognized and degraded by the SCF^COI1^ complex, comprising the subunits Skip, Cullin, and the F-box protein CORONATINE INSENSITIVE 1 (COI1), thereby relieving their inhibition of various transcription factors [107]. In apple, MdJAZ2 interacts with MdbHLH3, while MdJAZ1 interacts with Telomere-binding protein 1 (MdTRB1). JAZ protein degradation, releases MdbHLH3, enabling it to form activation complexes more effectively with MdMYB9 and MdMYB11; at the same time, JAZ degradation releases MdTRB1, enabling it to promote *MdMYB9* transcription [63,108]. Additionally, the Sucrose-nonfermenting 1-related protein kinase 1.1 (MdSnRK1.1) participates in sucrose-induced PA accumulation (see below), in part by phosphorylating and disrupting MdJAZ18 stability [109].

Salicylic acid (SA), as a key signal produced in response to pathogen invasion, can also promote PA biosynthesis. In black cottonwood, inoculation with the rust pathogen *Melampsora larici-populina* or SA application induces expression of the PA biosynthesis-promoting transcription factor gene *PtMYB134* in leaves, thereby significantly increasing accumulation of (+)-catechin and PA [110].

Light is a critical environmental factor regulating PA biosynthesis; changes in light quality (wavelength) and quantity (light intensity) exert effects through dedicated photoreceptors and signaling pathways. Ultraviolet B (UV–B) radiation is a stress that strongly induces PA biosynthesis. In poplar, UV irradiation significantly activates *MYB134* expression and that of its downstream target genes in the PA pathway [111]. In *Arabidopsis*, UV-B also promotes anthocyanin accumulation by inhibiting transcription of the repressor gene *MYBL2* [112].

The effects of light on PA content are more complex in developing grapes. Shading young fruits to block most visible and UV light significantly diminishes the PA content and degree of polymerization of PAs in the pericarp and downregulates expression of several PA biosynthetic genes, including *VvMYBPA1*, *VvLAR1*, and *VvANR*. However, blocking UV light alone resulted in lower flavonol biosynthesis and expression levels for the associated genes (such as *VvFLS4*), but had little effect on PA content. This finding indicates that in grape fruits, visible light rather than UV light is the main light signal that regulates PA biosynthesis, while UV light specifically regulates the flavonol branch of the pathway [113]. Light signaling factors such as LONG HYPOCOTYL 5 (MdHY5) integrate light signals with the PA biosynthetic pathways by activating downstream transcriptional cascades such as MdHY5→MdNAC52→MdMYB9/11 [114].

Mechanical wounding signals and high sugar levels are also integrated into the PA regulatory network. Mechanical wounding (such as leaf injury) strongly induces *MdMYB9* and *MdMYB11* expression in apple [63]. In poplar, wounding similarly induces *MYB134* expression and PA accumulation [111], possibly representing a proactive defense against potential herbivore attack. Treating the leaves of apple trees with sucrose promotes anthocyanin and PA accumulation, a response that involves the kinase MdSnRK1.1. This kinase transmits sugar abundance signals to the PA biosynthetic pathway through various mechanisms, including by regulating JAZ protein stability [109]. In tea plants, sucrose can also induce *CsMYB5b* expression, thereby promoting PA generation [80].

## 4 Current and potential applications of PAs

**Fig. 3:**
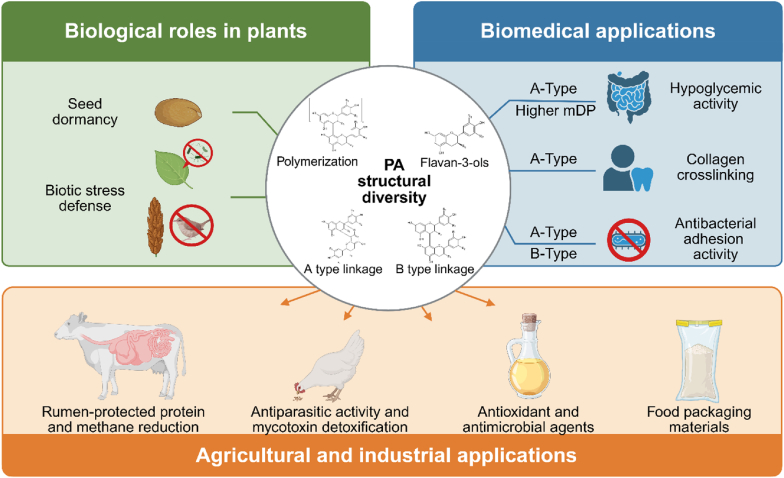
The structural diversity of PAs as a driver of their functional diversity and wide range of applications. The multiple applications of PAs are rooted in their extreme structural heterogeneity, encompassing variations in monomer composition, linkage types, and mean degree of polymerization (mDP). This structure-guided framework operates across three major domains: *in planta* roles, biomedical applications, and agricultural or industrial uses. In plants, PAs provide essential defense barriers for seed dormancy and protection against biotic stresses such as pathogens and herbivores. For human health, specific structural features determine targeted bioactivities. For example, A-type linkages and higher mDP provide hypoglycemic effects and promote collagen crosslinking in dentin, while B-type linkages contribute to cardiovascular protection. In agriculture and industry, structural parameters such as mDP and linkage type influence the rate of protein digestion in the rumen and methane production in livestock, as well as underlying applications as natural preservatives, antimicrobial agents, and functional enhancers in food packaging materials.

PAs have diverse applications—both current and potential—that reflect the activities conferred by their chemical structures, such as powerful antioxidant, protein-binding, and metal-chelating properties. However, converting natural PAs into stable, effective products faces a fundamental challenge: PAs are present in plant cells as mixtures with varying mDP, linkage patterns, substituent groups, and monomer composition (Fig. 3). Developing PA applications may therefore require switching from the use of plant crude extracts of variable composition to rational design targeting specific PA structures. This section will systematically analyze the state of current PA applications and future directions in different fields.

### 4.1 Medicine

Multiple studies have reported that PAs can inhibit carbohydrate-digesting enzymes, with some observing a possible relationship between PA structure and this activity. For example, PAs extracted from Chinese firethorn (*Pyracantha fortuneana*) and bird cherry (*Prunus padus*) inhibit α-glucosidase and α-amylase activity [47,[115], [116], [117]]. Some studies suggest that A-type linked PAs generally exhibit superior inhibitory activity compared to those with B-type linkages, spontaneously binding to α-glucosidase and altering enzyme conformation [12]. Additionally, *A. melanocarpa* PA fractions with high mDP had stronger inhibitory activity than those with low mDP, suggesting that the strength of inhibition positively correlates with mDP (Table 1) [46].

**Table 1:** Representative relationships between PA structure and activity.

Structural feature	Application	Mechanism of action	Structural comparison	References
Epigallocatechin-(4β→6)-epigallocatechin-(2β→O→7,4β→8)-catechin (A-type trimer)	Hypoglycemic activity	Binds to α-glucosidase through hydrophobic interactions, altering enzyme conformation and reversibly inhibiting its activity in a mixed-type manner	This A-type trimer shows the strongest α-glucosidase inhibition (IC50 = 25.28 ± 0.67 μg/mL); A-type linkages are generally more effective than B-type linkages	[12]
A-type PA trimers and tetramers	Dentin biomodification	A-type linkages introduce additional covalent bonds that restrict molecular rotation, enabling efficient and stable collagen crosslinking	B-type linked PAs show lower binding efficiency due to greater molecular flexibility	[118]
B-type PAs (mDP >10)	Antibacterial coating for biomaterials	Alters bacterial surface hydrophobicity, effectively inhibiting adhesion of Gram-positive bacteria (e.g., *S. aureus*)	B-type PAs preferentially inhibit adhesion of Gram-positive bacteria, while A-type PAs preferentially inhibit adhesion of Gram-negative bacteria	[119]
A-type PAs (mDP 4–10)	Anti-adhesion against uropathogenic bacteria	Inhibits adhesion of P-fimbriated *E. coli* to uroepithelial cells	A-type linkages are essential for the anti-adhesion activity; B-type PAs lack this effect	[120]
High mDP PAs (mDP = 73)	Lowering lumen methane production	Inhibits ruminal methanogenesis	Higher mDP correlates with higher inhibition of methane production	[121]
5-Deoxyflavan-3-ol-based PAs	Lowering lumen methane production	Unique chemical stability in rumen environments confers strong inhibition of methane production despite moderate antioxidant activity	Nonlinear relationship between antioxidant activity and inhibition of methane production; structural type rather than antioxidant capacity determines efficacy	[122]

PAs also have potential applications in oral health, as dental biomaterials with collagen crosslinking activity. For example, PAs with A-type double-bond linkages, such as (+)-epigallocatechin-(4β→8)-epigallocatechin-(2β→O→7,4β→8)-catechin, have been shown to modify the dentin in teeth (Table 1) [123]. Compared to B-type linkages, A-type linkages confer greater structural rigidity to PAs, and a higher proportion of double bonds is associated with a stronger interaction with dentin type I collagen [118].

PAs also have antibacterial and anti-inflammatory properties. Commercial pine bark extract, which mainly contains PAs with mDP 2–7, exhibits broad-spectrum antifungal activity against various yeasts [119]. Similarly, pine leaf–derived PAs specifically inhibit the adhesion of *Staphylococcus aureus* and other Gram-positive bacteria to the surface of biomaterials used in medical applications, while they have negligible effects on Gram-negative bacteria like *Pseudomonas aeruginosa* [119]. These findings provide a basis for developing PA-based topical antibiotics. A new hydrogel wound dressing was recently formulated to harness the antioxidant and antibacterial properties of PAs, composed of alginate, graphene oxide, and condensed tannins. This material supported tissue regeneration [124], marking an important step for PAs in the development of innovative medical materials.

In summary, current evidence suggests that different targets for medical applications may require different PA structures (Fig. 3). In future, the development of new targeted drugs or functional foods will require substantial work to clarify the feasibility and necessity of screening for or customizing specific PA components.

### 4.2 Food industry and packaging materials

PAs also have potential applications in the food industry as natural additives. Natural PAs can be used directly, for example in emulsions that exploit the interaction between proline-rich gliadin and PAs, while also retaining the antioxidant and antibacterial activities of PAs [125]. Chemical modifications of PAs are another option. In one example, PAs were deployed to inhibit heterocyclic amine formation in fried foods. To address the limited solubility of oligomeric PAs (mDP <10) in oil, lipophilic derivatives of PAs were prepared by introducing stearic acid chains. Preliminary results showed enhanced antioxidant efficiency of the modified PAs relative to unmodified PAs in oil–fat systems [126]. Although the practical application of these modified PAs requires further evaluation, this study demonstrates the potential for directionally optimizing the physicochemical properties of PAs through chemical modification.

In food packaging, PAs have been extensively studied as functional additives that enhance the mechanical properties, UV and thermal stability, and gas barrier performance of packaging polymers, while also extending food shelf life by retarding oxidative degradation. The phenolic hydroxyl groups of PAs can form hydrogen-bond networks with polymer matrices such as polylactic acid, chitosan, and polyolefins, enhancing the mechanical strength, barrier properties, and stability of these materials [2]. The antioxidant and antibacterial activities of PAs may also help extend food shelf life [9,127]. However, the structural heterogeneity of natural PAs poses a core challenge for this application; variations in the mDP and monomer composition (catechin/epicatechin ratio and B-ring hydroxylation pattern), linkage types (A-type vs B-type), and chemical modifications (such as galloylation) directly affect PA compatibility with polymers and crosslinking density, causing large batch-to-batch variations in material performance. Therefore, achieving standardized production depends on developing technologies to fractionate crude PA extracts into structurally homogeneous fractions (e.g., by mDP range or linkage type) through methods such as size-exclusion chromatography or selective precipitation, enabling consistent material performance (Fig. 3) [9].

### 4.3 Animal nutrition and the feed industry

In ruminant animal nutrition, the effects of PA application are complex, often representing a balance between improved nitrogen utilization efficiency versus improved digestibility.

The presence of PAs in feed can have multiple benefits. By diminishing methane emissions and protecting proteins from digestion in the rumen, PAs improve nitrogen utilization efficiency and lower bloat risk, although these benefits vary in practice. The effects of PAs may occur via more than one mechanism. For example, some studies showed that PAs with a high mDP may be more effective than PAs with a lower mDP (Table 1) [121]. Additionally, systematic comparison across multiple legumes uncovered a strong nonlinear association between the *in vitro* antioxidant activity of PAs and their ability to inhibit methane production, but conventional structural parameters (monomer composition, stereochemistry, galloylation, molecular weight) showed no predictive value [122]. This finding suggests that the mechanisms behind lower methane emissions may not be limited to direct effects on the microorganisms in the rumen but may also involve interference with oxidation-reduction reactions. More importantly, specific chemical structures may be more critical than total PA content or average mDP. For instance, PAs from prairie acacia (*Acacia angustissima*) have an extremely strong capacity to minimize methane emissions due to their unique 5-deoxyflavan-3-ol structures, suggesting that their chemical stability may be a decisive determinant of their efficacy in rumen environments (Table 1) [122].

At optimal levels and composition, PAs improve nitrogen utilization by selectively binding to soluble proteins in feed; this produces complexes that prevent excessive microbial degradation of proteins in the rumen, allowing them to reach the hindgut for uptake, forming complexes that prevent excessive microbial degradation of proteins in the rumen, which then dissociate in the acidic lower gut to release amino acids for absorption. However, when PA dosage is too high or when PAs have excessively reactive structures, they can indiscriminately bind to feed proteins, rumen microbial enzymes, and other nutrients. This can inhibit rumen microbial activity and diminish total dry matter digestibility, negatively influencing animal production performance [128]. Therefore, improving the efficiency of nitrogen utilization must be balanced against forage digestibility (Fig. 3).

Other potential benefits of PAs in feed are that they can be antiparasitic, such as against gastrointestinal nematodes, and they can protect against food poisoning, such as from aflatoxin B1 [129,130].

### 4.4 Technical challenges in characterizing PA structure and structure–function relationships

The structural complexity of PAs poses formidable analytical challenges that no single method can fully resolve. Thiolytic degradation coupled with HPLC-MS, a technique used to determine PA degree of polymerization and monomer composition, is limited to B-type PAs with conventional 4 → 8 or 4 → 6 interflavan bonds and fails to digest chemically resistant 5-deoxy PAs [122]. Nuclear magnetic resonance (NMR) spectroscopy provides stereochemical detail but requires low-temperature acquisition (<270 K) for B-type PAs because rapid conformational changes at room temperature blur the NMR signals; cooling slows molecular motion and sharpens the spectra. In addition, signals from terminal units diminish with higher DP [118]. Matrix-assisted laser desorption/ionization–time-of-flight mass spectrometry (MALDI-TOF MS) reveals subunit composition and DP distribution but loses ionization efficiency at DP values above 12 and cannot resolve stereochemistry [45]. These limitations are not insurmountable: combining complementary techniques such as thiolysis–high-performance liquid chromatography with diode-array detection (HPLC–DAD), 2D heteronuclear single quantum coherence (HSQC) NMR, and Fourier-transform–ion cyclotron resonance (FT–ICR) MS, provides convergent structural evidence sufficient for most research purposes [45]. The key question is therefore not whether PA structures can be characterized, but what level of resolution is required in any given context.

Establishing relationships between the structure of PAs and their activity is equally difficult because multiple structural parameters co-vary in natural PA mixtures. Critically, different applications demand different levels of structural precision. In the pharmaceutical field, where PAs serve as specific bioactive agents at small doses (e.g., α-glucosidase inhibitors, dentin crosslinkers), high-resolution structural characterization is essential, but the relatively small quantities required for bioassay-guided discovery and early-stage development make preparative purification feasible. In ruminant nutrition, effective mitigation of methane emissions requires PA levels of at least 6 g/kg feed dry matter; at such a large scale, structural homogeneity is impractical, yet population-level parameters such as mDP range have been sufficient to explain biological variation and guide formulation [121,122]. For food packaging and industrial materials, where performance depends on bulk properties reflecting phenolic hydroxyl density, molecular weight distribution, and antioxidant capacity, batch-to-batch standardization of these aggregate parameters is more practical than full structural elucidation [2]. Thus, matching analytical depth to application requirements will be essential for efficiently translating PA research into practical outcomes.

## 5 Conclusions and future prospects

This review systematically examined recent progress in the study of PAs, from their chemical structure, biosynthesis, and regulatory networks to their potential applications. The potential functions of PAs are as diverse as their structures, which are precisely regulated from gene to ecosystem levels. The MBW complex–centered regulatory framework is remarkably conserved and provides flexible adaptive potential across species. However, translating the tremendous potential of PAs into real-world applications still faces multiple challenges. Meeting these will require flipping the existing research paradigm: future studies should start with defining the desired PA functions, then identifying the structures that will fulfill those functions and, finally, developing innovative technologies for production and preparation of the target PAs.

Several key priorities emerge for future PA research. First, the field urgently needs to shift from “crude extract” thinking to “structurally defined” thinking, as different applications require different PA structures. Defining “ideal PA structure” blueprints for different research fields, both fundamental and applied, should be guided by studies of the precise structure–activity relationships of PAs. Second, new technologies are needed for producing and refining target PA structures; these could include improved fractionation methods for purifying PAs and metabolic engineering to favor production of target PAs, either through editing of existing plant genes or through *de novo* biosynthesis via synthetic biology in a microbial or plant chassis.

Although the MBW transcriptional complex has been widely confirmed as a core regulatory module of PA biosynthesis, a considerable gap remains in translating this knowledge into practical applications. Future breakthroughs lie in elucidating the upstream signals that modulate MBW complex activity under specific circumstances and in exploiting emerging technologies for programmable regulation of PA biosynthesis. These include the use of deep learning in two respects: first, to design synthetic transcription factors that can activate downstream PA biosynthetic genes independently of the MBW complex; and second, to train models on large datasets of promoter sequences and gene expression patterns across different tissues and conditions, enabling the identification of novel *cis*-regulatory elements and prediction of the effects of targeted promoter modifications. Such predictive insights, combined with CRISPR-based promoter editing technologies, could enable programmable regulation of PA biosynthesis, though substantial validation will be required before practical implementation.

The most central remaining challenges are to elucidate PA transport and to understand the mechanisms that determine PA structure. mDP and linkage types are keys to the functional differences among PAs, but the higher-order structures of PAs likely depend on non-enzymatic polymerization reactions. Where PA polymerization occurs within cells—whether as proposed by the tannosome-shuttle model or by the membrane transporter model—must be clarified before we can fully understand how mDP and linkage type are regulated. Future research urgently needs high spatiotemporal resolution live-cell imaging techniques and *in situ* chemical probes to track PA precursor transport and polymerization dynamics in real time.

## CRediT authorship contribution statement

**Yi Qiao:** Writing – original draft. **Pan Zhen:** Resources. **Qian Gao:** Resources. **Feifei Yu:** Supervision, Resources. **Qi Xie:** Writing – review & editing, Supervision. **Jiayang Shi:** Writing – review & editing, Funding acquisition.

## Declaration of competing interest

The authors declare that they have no known competing financial interests or personal relationships that could have appeared to influence the work reported in this paper. Qi Xie was not involved in the peer review process of the manuscript.

## Data Availability

No datasets are associated with this review.
