# Supplementary material for: Proanthocyanidins: structure, biosynthesis, regulation, and structure–activity relationships
Source: aBIOTECH. 2026 Mar 31;7(3):100047. doi: 10.1016/j.abiote.2026.100047 (PMC13136753; doi:10.1016/j.abiote.2026.100047)
Supplement: Multimedia component 1 [file mmc1.docx]

# Supplemental Table S1. Comparison of Proanthocyanidin Structural Features in Different Plant Species

| Species | Tissue | mDP / Main DP Range | Main Constituent Units | Main Linkage Type | Galloylation Degree | Main References |
| --- | --- | --- | --- | --- | --- | --- |
| Grape (*Vitis vinifera*) | Seeds | main DP range: 2.3–30.3 | (+)-catechin,  (−)-epicatechin,  (−)-epicatechin gallate | mainly B-type | different varieties: 13.1-32.2% | [8,131,132] |
| Grape (*Vitis vinifera*) | Skin | different varieties mDP 2.1-85.7; *cv. Shiraz*: early development mDP=7.3, *maturity* mDP=27.0 | (+)-catechin,  (−)-epicatechin;  some varieties contain (+)-gallocatechin,  (−)-epigallocatechin | mainly B-type | different varieties: 1.4-19.0% | [8,131,132] |
| Apple (*Malus domestica*) | Fruit | main DP range: 3-10 | (+)-catechin,  (−)-epicatechin | B-type predominant (A-type bond oligomers only ~5%) | Not reported | [6] |
| Cranberry (*Vaccinium macrocarpon*) | Fruit | main DP range: 2-12 | (+)-catechin,  (−)-epicatechin | A-type predominant (A-type bond oligomers 96%) | Not reported | [6] |
| Peanut (*Arachis hypogaea*) | Seed coat / Roasted skin | main DP range: 2-12 | mainly (+)-catechin,  (−)-epicatechin;  contain minor amounts of  (+)-gallocatechin,  (−)-epigallocatechin | A-type predominant (A-type bond oligomers 96%) | Not reported | [6,133] |
| Cocoa (*Theobroma cacao*) | Seeds | main DP range: 3-5 | (−)-epicatechin | all B-type | Not reported | [48] |
| Plum (*Prunus salicina*) | Peel | mDP=5.3; main DP range: 2-13 | (−)-epicatechin | A-type, B-type | Not reported | [134] |
| Bird cherry (*Prunus padus*) | Fruit | mDP=5.6 | (+)-catechin,  (−)-epicatechin predominant;  (+)-gallocatechin,  (−)-epigallocatechin | A-type, B-type | Not reported | [47] |
| Black chokeberry (*Aronia melanocarpa*) | Fruit | mDP:=49.3 ± 3.8 | (−)-epicatechin predominant; (+)-catechin trace amounts | Not reported | Not reported | [46] |
| Mangosteen (*Garcinia mangostana*) | Peel | mDP=6.6; main DP range: 2-12 | mainly (−)-epicatechin, contains minor amounts of (+)-catechin, (+)-afzelechin, (+)-gallocatechin | mainly B-type linkages, contains minor A-type linkages | Not reported | [135] |
| Lychee (*Litchi chinensis*) | Peel / Seed | mDP=4 | (−)-epicatechin | A-type >60% | Not reported | [136-138] |
| Persimmon (*Diospyros kaki*) | Fruit | mDP=26 | epigallocatechin gallate, epicatechin gallate,  (+)-gallocatechin,  (−)-epigallocatechin | A-type/B-type | ~72% | [49] |
| Persimmon (*Diospyros kaki*) | Peel | mDP=10.18 | gallocatechin gallate,  epigallocatechin gallate,  catechin gallate,  epicatechin gallate,  (+)-catechin,  (−)-epicatechin | mainly A-type | >74.79% | [139] |
| Persimmon (*Diospyros kaki*) | Leaves | mDP=6.83±0.57 | mainly: (+)-catechin;  secondary: (+)-gallocatechin,  catechin gallate | mainly: B-type; secondary: A-type | Partial galloylation | [140] |
| Saskatoon berry (*Amelanchier alnifolia*) | Fruit | main DP range: 5-10 | mainly (−)-epicatechin; contains minor amounts of (+)-catechin | B-type linkages predominant, A-type linkages also detected | Not reported | [141] |
| Rabbiteye blueberry (*Vaccinium virgatum*) | Leaves | mDP: 3.9 (April) → 8.0 (October) | (−)-epicatechin predominant | A-type, B-type | Not reported | [142] |
| Rabbiteye blueberry (*Vaccinium virgatum*) | Branches | mDP: ~4 (stable year-round) | (+)-catechin,  (-)-epicatechin | A-type, B-type | Not reported | [142] |
| Cinnamon (*Cinnamomum spp*.) | Bark | *C. cassia* & *C. japonica*: trimers/tetramers; *C. verum*: mDP=5.2 | *C. verum*: (+)-catechin;  (-)-epicatechin | *C. cassia*: B-type; *C. japonica*: A-type; *C. verum*: 79% B-type, 21% A-type | Not reported | [143,144] |
| Masson pine (*Pinus massoniana*) | Bark | trimers, tetramers, hexamers | (+)-catechin,  (-)-epicatechin,  (+)-afzelechin ,  (-)-epiafzelechin | A-type, B-type | No modification detected | [4] |
| Ephedra (*Ephedra spp.*) | Stems / Aerial parts | main DP range: 2-5 | (+)-catechin,  (-)-epicatechin,  (+)-gallocatechin,  (-)-epigallocatechin | mainly A-type | Not reported | [145-147] |
| Chinese banyan (*Ficus altissima*) | Bark | mDP: 2.4, 6.6, 10.5, 13.4 (four fractions) | (+)-catechin,  (−)-epicatechin predominant, (+)-gallocatechin,  (−)-epigallocatechin | B-type | Not reported | [148] |
| Chinese bayberry (*Myrica rubra*) | Bark | mDP=5.22 | (+)-gallocatechin,  gallocatechin gallate | B-type | Galloylation present | [149] |
| Loquat (*Eriobotrya japonica*) | Leaves | mDP=7.25±0.93 | (+)-catechin,  (+)-afzelechin,  (+)-gallocatechin,  gallocatechin gallate, | mainly: B-type; secondary: A-type | Partial galloylation | [140] |
| Pu'an tea (*Camellia sinensis var. puanensis*) | Leaves | dimers, trimers | (+)-catechin,  (-)-epicatechin | B-type | Partial galloylation | [150] |
| Barrel medic (*Medicago truncatula*) | Seeds | mDP: 10 | (−)-epicatechin | Not reported | Not reported | [44] |
| Neptunia lutea | Leaves | mDP=11.5 (thiolysis), 8.1 (NMR) | (-)-epigallocatechin | B-type | 32.0% (thiolysis), 34.4% (NMR) | [45] |
| Various clovers (*Trifolium spp.*) | Flowers | mDP range: 8.6-13.9 | (+)-catechin,  (-)-epicatechin,  (+)-gallocatechin,  (-)-epigallocatechin | Not reported | Not reported | [151] |
| Selliguea feei | Rhizome | Not reported | (+)-afzelechin,  (-)-epiafzelechin | A-type, B-type | Not reported | [152] |

Note: mDP = mean degree of polymerization; DP = degree of polymerization. Data compiled from various sources as cited in the references column. Some entries contain multiple measurements from different cultivars or developmental stages.

**References**

[131] Hayasaka Y, Waters EJ, Cheynier V, Herderich MJ, Vidal S. Characterization of proanthocyanidins in grape seeds using electrospray mass spectrometry. Rapid Commun Mass Spectrom 2003;17(1):9–16.

[133] Kyraleou M, Kotseridis Y, Koundouras S, Chira K, Teissedre PL, Kallithraka S. Effect of irrigation regime on perceived astringency and proanthocyanidin composition of skins and seeds of *Vitis vinifera* L. cv. Syrah grapes under semiarid conditions. Food Chem 2016;203:292–300.

[133] Monagas M, Garrido I, Lebrón-Aguilar R, Gómez-Cordovés MC, Rybarczyk A, Amarowicz R, et al. Comparative flavan-3-ol profile and antioxidant capacity of roasted peanut, hazelnut, and almond skins. J Agric Food Chem 2009;57(22):10590–9.

[134] Zhang L, Zhang H, Tang L, Hu X, Xu M. Isolation, characterization, antioxidant activity, metal-chelating activity, and protein-precipitating capacity of condensed tannins from plum (*Prunus salicina*) fruit. Antioxidants (Basel) 2022;11(4):714.

[135] Fu C, Loo AE, Chia FP, Huang D. Oligomeric proanthocyanidins from *mangosteen pericarps*. J Agric Food Chem 2007;55(19):7689–94.

[136] Gong Y, Fang F, Zhang X, Liu B, Luo H, Li Z, et al. B type and complex A/B type epicatechin trimers isolated from litchi pericarp aqueous extract show high antioxidant and anticancer activity. Int J Mol Sci 2018;19(1):301.

[137] Miranda-Hernández AM, Muñiz-Márquez DB, Wong-Paz JE, Aguilar-Zárate P, de la Rosa-Hernández M, Larios-Cruz R, et al. Characterization by HPLC-ESI-MS2 of native and oxidized procyanidins from litchi (*Litchi chinensis*) pericarp. Food Chem 2019;291:126–31.

[138] Xie C, Wang K, Liu X, Liu G, Hu Z, Zhao L. Characterization and bioactivity of A-type procyanidins from litchi fruitlets at different degrees of development. Food Chem 2023;405(Pt A):134855.

[139] Ye H, Luo L, Wang J, Jiang K, Yue T, Yang H. Highly galloylated and A-type prodelphinidins and procyanidins in persimmon (*Diospyros kaki* L.) peel. Food Chem 2022;378:131972.

[140] Tao W, Pan H, Jiang H, Wang M, Ye X, Chen S. Extraction and identification of proanthocyanidins from the leaves of persimmon and loquat. Food Chem 2022;372:130780.

[141] Jin AL, Ozga JA, Kennedy JA, Koerner-Smith JL, Botar G, Reinecke DM. Developmental profile of anthocyanin, flavonol, and proanthocyanidin type, content, and localization in saskatoon fruits (*Amelanchier alnifolia* Nutt.). J Agric Food Chem 2015;63(5):1601–14.

[142] Koga Y, Setoguchi Y, Sugamoto K, Goto Y, Hirano T, Kunitake H. Seasonal variation and mean degree of polymerization of proanthocyanidin in leaves and branches of rabbiteye blueberry (*Vaccinium virgatum Aiton*). Plants (Basel) 2024;13(13):1864.

[143] Lu Z, Jia Q, Wang R, Wu X, Wu Y, Huang C, et al. Hypoglycemic activities of A- and B-type procyanidin oligomer-rich extracts from different Cinnamon barks. Phytomedicine 2011;18(4):298–302.

[144] Williams AR, Ramsay A, Hansen TV, Ropiak HM, Mejer H, Nejsum P, et al. Anthelmintic activity of trans-cinnamaldehyde and A- and B-type proanthocyanidins derived from cinnamon (*Cinnamomum verum*). Sci Rep 2015;5:14791.

[145] Barreiros AL, David JP, de Queiroz LP, David JM. A-type proanthocyanidin antioxidant from *Dioclea lasiophylla*. Phytochemistry 2000;55(7):805–8.

[146] Zang X, Shang M, Xu F, Liang J, Wang X, Mikage M, et al. A-type proanthocyanidins from the stems of *Ephedra sinica* (Ephedraceae) and their antimicrobial activities. Molecules 2013;18(5):5172–89.

[147] Orejola J, Matsuo Y, Saito Y, Tanaka T. Characterization of proanthocyanidin oligomers of *Ephedra sinica*. Molecules 2017;22(8):1308.

[148] Chai W, Wu Y, Li X, Zeng S, Cheng Y, Jiang W, et al. Relationships between degree of polymerization and activities: A study on condensed tannins from the bark of *Ficus altissima*. Int J Biol Macromol 2024;274(Pt 1):133306.

[149] Xia S, Wei Z, Kong X, Jia B, Han S. Antioxidative properties of bayberry tannins with different mean degrees of polymerization: Controlled degradation based on hydroxyl radicals. Food Res Int 2022;162(Pt B):112078.

[150] Li YF, Ouyang SH, Chang YQ, Wang TM, Li WX, Tian HY, et al. A comparative analysis of chemical compositions in *Camellia sinensis* var. *puanensis Kurihara*, a novel Chinese tea, by HPLC and UFLC-Q-TOF-MS/MS. Food Chem 2017;216:282–8.

[151] Meagher LP, Widdup K, Sivakumaran S, Lucas R, Rumball W. Floral Trifolium proanthocyanidins: polyphenol formation and compositional diversity. J Agric Food Chem 2006;54(15):5482–8.

[152] Fu C, Wang H, Ng WL, Song L, Huang D. Antioxidant activity and proanthocyanidin profile of *Selliguea feei* rhizomes. Molecules 2013;18(4):4282–92.
